# Towards the engagement of citizens in SOSTs decision-making: participatory models setting a common ground for border surveillance and respect of fundamental rights. Findings from ARESIBO H2020 project

**DOI:** 10.12688/openreseurope.15079.1

**Published:** 2023-01-25

**Authors:** Riccardo Laterza, Daniele Del Bianco, Ramona Velea, Olivia Ferrari, Lorenzo De Sabbata, Ezio Benedetti

**Affiliations:** 1 Institute of International Sociology of Gorizia – ISIG, Gorizia, 34170, Italy; 2 Department of Social and Political Sciences, University of Trieste, Trieste, 34127, Italy

**Keywords:** Participation, border surveillance, participatory models, Surveillance-Oriented Security Technologies (SOSTs) for borders control, citizens’ involvement, privacy, human rights, methodology.

## Abstract

ARESIBO is an H2020 project aiming to improve the efficiency of border surveillance systems by providing the operational teams, as well as the tactical command and control level with accurate and comprehensive information related to border control by different perspectives. Human Rights Groups’ (HRGs) and Civil Society Organisations’ (CSOs) involvement and participation in decision making related Surveillance-Oriented Security Technologies (SOSTs) for border surveillance is considered as a key-factor to ensure the coexistence of two only apparently opposite needs: security and respect of fundamental rights (i.e., privacy, refugees etc.). Starting from this general approach, the paper represents the second part of a research diptych dedicated to present the main achievements and methods proposed by ARESIBO to enhance participation in border surveillance. It outlines the ARESIBO Participatory Model (APM) as a tool to improve HRGs, CSOs, and ultimately citizens’ involvement related to border surveillance in general and the adoption of specific SOSTs for borders’ control. The first part of the paper introduces some key elements included in the ARESIBO desk research that led to the elaboration of the APM (i.e., literature review, semantic analysis), describing who were the targets of that approach (i.e., *stakeholders, end-users, actors*). After a contextualization on the three interlaced dimensions of surveillance, security and privacy related borders’ control, the paper presents the three components of the methodological framework within which this citizens’ involvement process has been developed (i.e., ARESIBO Participation Framework, ARESIBO Participation Strategy, ARESIBO Participation Action Plan). In the conclusions, by outlining the first partial application of the model within the ARESIBO framework, the paper argues that APM can represent a pilot for a more structured and duplicable participatory model, also through additional research regarding participatory models applied to SOSTs development processes.

## 1 Introduction

Borders are an abstract concept and a reality at the same time. As stated in Art. 19 of the United Nations (UN) Universal Declaration of Human Rights: « *Everyone has the right to freedom of opinion and expression; this right includes freedom to hold opinions without interference and to seek, receive and impart information and ideas through any media and regardless of frontiers*». Most of International Organisations (IOs) dealing with fundamental rights and freedoms ^1^ stress that human rights and borders’ management are two dimensions that are strictly interlaced. The International Organisation for Migrations (IOM) and the United Nations High Commissioner for Refugees (UNHCR) agree that a better and more structured involvement of and cooperation with Human Rights Groups (HRGs) and Civil Society Organizations (CSOs) working on borders’ matters is of paramount importance to guarantee the respect of these rights at all levels ^2^. This approach is followed also by European Union (EU) institutions and agencies, whereas the most relevant EU agency dealing with external borders control (i.e., FRONTEX) stressed on several occasions the centrality of human rights and the necessity to enhance the participation of CSOs and HRGs in decision making processes related to border management ^3^.

By this point of view, it is not relevant to consider borders only as linear and static expressions of state power. A more systematic and sophisticated approach is needed to understand their complexities. Borders have been conceptualized in terms of « *dynamic “bordering” processes socially performed at both practical and discursive levels*» (see Van Houtum & Van Naerssen, 2002). Border performativity highlights how borders are continually made and re-made through three « *performative registers*»: state description of borders, policing and patrolling of borders, and popular support and contestation over borders ( Johnson *et al*., 2011). The current analysis will focus its attention on the third register in particular, whereas the role of ordinary people (including HRGs and CSOs) in « *constructing, shifting, or even erasing borders*» is deemed important ( Rumford, 2012, p. 897).

The issue of EU’s external borders control and management, as well as the more specific field of analysis related to the technical/technological solutions adopted by EU member States and EU agencies ^4^ to guarantee the security of these borders, have a strong and direct connection with the respect of human rights and fundamental freedoms ( Carrera & Stefan, 2021), both in terms of refugees/migrants’ and citizens’ rights ( Costello, 2020). In fact, since mid-2015 a series of problems and shortcomings related the management of migrations and borders came to light and the EU’s border management policy had to adapt its approach to the exceptional arrival of refugees and migrants (regular and irregular). On one hand, it became immediately clear that a human rights-based approach was necessary to guarantee the respect of fundamental rights of these individuals by police and border authorities. On the other hand, this exceptional situation, combined with the more recent impact of coronavirus disease (COVID-19) pandemic, led to the implementation of new approaches and technologies for the control of EU borders. That implied the need to (re)consider the impact these innovative technological solutions for borders’ control have on the fundamental rights both of migrants/refugees/asylum seekers and of EU citizens, who live in border areas or cross EU borders for different reasons (tourism, work, etc.).

Starting from these general considerations, this paper represents the second part of a research diptych stemming from the activities and the results of the H2020 ARESIBO project ^5^. It is the logical continuation of the paper “The missing piece. The perceived marginalization of CSOs and HRGs in SOSTs’ decision-making processes: a systematic review” also elaborated in the framework of ARESIBO project.

Stemming from these results, this (second) paper introduces a heuristic framework aimed at understanding the potentials of the involvement of HRGs and CSOs in border surveillance decision making, thus providing a concrete proposal to address the necessity expressed by IOs and EU to enhance their participation on these matters, so to foster societal acceptance of specific SOSTs by selected targets/groups. More specifically, the paper illustrates the main results achieved by a specific activity implemented in the frame of the ARESIBO project: the elaboration of a the **ARESIBO Participatory Model** – hereinafter **APM**.

The primary goal of APM model is to gather data on citizens’ needs and perceptions of the potential societal effects and technical impact of SOSTs, through CSOs and HRGs involvement. The secondary scope is the integration of such information in the design and development process of SOSTs. This essay presents the structure, the logic and the functioning of the APM, proposing the approach included in the participatory model as a key factor to ensure the involvement of local communities, EU citizens, HRGs and CSOs in border management decisions.

The research focuses first on the methodology used to identify the main scientific literature dedicated to this argument (see par. 2), presenting the targets of that approach, analyzing the strong connection existing between the three interlaced dimensions of surveillance, security and privacy related borders’ control, and introducing the main findings and the methodology of the desk research activities – based on both semantic analysis and literature review – that led to the elaboration of the APM. Specific attention (see par. 3.1) is dedicated to the potential subjects of the involvement process (i.e., targets of involvement: *stakeholders, end-users* and *actors*).

The analysis presents the main features characterizing the involvement activities, both in terms of tools and procedures, as follows:

- Methods of involvement and/or participation of *stakeholders, actors* and *end-users* ( par. 3.1.1.2, par. 3.1.2.2 and par. 3.1.3.2).
- The context in which APM has been developed, that is border security-oriented surveillance technologies ( par 3.2).
- The main elements included in the practical elaboration of the APM which is structured: **ARESIBO Participation Framework** (APF) – the conceptual framework within which the targets and methods of involvement are defined ( par. 3.3.1); **ARESIBO Participation Strategy** (APS) – detailing the goals and specific objectives of involvement for a specific context (i.e., ARESIBO pilot communities – par. 3.3.2).; **ARESIBO Participation Action Plan** (APA) – detailing the operational steps and tools to be implemented by partners in the involvement process, as well as the tools for the monitoring and evaluation of such process ( par. 3.3.3).

The conclusions argue that the APM, partially deployed within ARESIBO project, can represent a pilot model for a more structured and replicable participatory model . In other words, the APM can be duplicated and further validated/developed in other similar contexts (e.g. research projects, or at an operational level in connection with consultative mechanisms of EU agencies).

## 2 Methods

The first step made towards the elaboration of the APM has been the definition of the targets of involvement as the potential subjects of the participatory process. Four categories of targets have been initially identified: *citizens, stakeholders, actors*, and *end-users*. However, out of the four main preliminary identified targets, only three have been further analysed: *stakeholders, actors,* and *end-users*. ^6^

Therefore, the research questions that section 3 addresses are the following: What are the primary analytic contexts in which the terms of the inquiry are often framed? What are the common definitions for each term, and what practical applications do those definitions suggest? What are the primary classification schemes supporting the analysis suggested in the sources?

To do so, the paper presents:

- A semi-systematic review ( Snyder, 2019) of relevant academic literature (papers, books, book sections and official documents) written in English and made digitally available through Google Scholar ( Gusenbauer, 2019) ^7^. A total of 336 sources of relevant academic literature have been collected applying two criteria: sources online and mentioning at least once the inquired term ^8^. To give a general picture of the concepts under investigation, a semantic analysis was carried out based on the academic sources, focusing on commonalities and variations of the terms in the selected literature ^9^.
- An analysis of the meaning attached to the investigated terms in EU official documents. These definitions were collected and discussed as a starting point for the development of the research. For the purpose of the study, the EU’s terminology database IATE has been searched ^10^.
- In order to explain how the words under investigation are further defined and practically applied in a scientific and technological context, the research also considered pertinent scientific and technical literature associated with the creation of EU initiatives and policies. Scientific and technical papers, reports and contributions – with the exception of licenses and patents – written in English and collected by the Joint Research Centre (JRC) Publications Repository ^11^ have been taken into consideration through a semi systematic review ( Snyder, 2019). To implement a relevance-informed criterion of selection, the first 7 results according to the JRC relevance rank have been selected and inquired in the light of the three abovementioned research questions.
- Finally, the research considered a more operational approach, by analysing the publicly available information and deliverables of EU funded projects – both concluded and still ongoing – in the field of security, looking for explicit or implicit definitions of the inquired terms. For this purpose, Seventh (FP7) and Eighth (Horizon 2020) Framework Programmes have been considered, by searching for financed projects through the CORDIS portal ^12^. The research focused on 82 sources (of which 25 were FP7 and 57 were H2020 projects) for EU funded projects review: 11 projects mentioning the term *stakeholders*, 3 mentioning *actors*, and 2 mentioning *user/end-users*.

Summarizing, the different approaches were grouped on three levels according to their focus on the inquired terms. While the academic research semantic analysis provides a general overview ( *level 1*), the analysis on the official definitions used in the context of EU policy-making processes adds information on the use of the inquired terms in their legal framework of reference ( *level 2*). Lastly, the combination of EU funded research review, academic research semi-systematic review and EU funded projects review adopts a more specific and detailed perspective on the definitions of inquired terms, as well as on their operationalisation in several fields of analysis and action ( *level 3*), thus underpinning a general-to-specific structure which characterize this part of the project.

After presenting the results of the desk research for each of the terms, relevant methods and tools to enhance the involvement of *stakeholders, actors,* and *end-users* are offered. That part is organised around three central research questions that are modified for each field under investigation:

- What are the contexts or techniques of action that are proposed and/or implemented in the sphere of action?
- What are the primary tools for involvement that have been studied, suggested, and/or whose implementation has been examined in the sources?
- What are the roles that *stakeholders, actors,* and *end-users* will play in organizing and/or putting into practice the techniques of involvement?

The three research questions are addressed for every inquired term ( *stakeholder, actor* and *end-user*) through the combination of the three-level methodology described above. First, the EU Funded Research Review, taking into consideration 15 sources in total: 4 sources for *stakeholders’* involvement, 6 sources for *actors’* participation and 5 sources for *end-users’* involvement. Second, the Academic Research Semi-Systematic Review, taking into consideration 19 sources: 7 sources for *stakeholders’* involvement, 6 sources for *actors’* participation and 6 sources for *end-users*’ involvement. Third, the EU Funded Projects Review approach, taking into consideration the EU funded projects aforementioned.

## 3 Results

### 3.1 Stakeholder, actor, and end-user: attached meanings and methodologies of involvement

***3.1.1 Stakeholder(s)***

**3.1.1.1 Meaning of the term**

The main results of the semantic analysis ( *level 1*) made for the term *stakeholder* individuated 26 nodes, each one representing a word with more than 4,000 occurrences in the selected literature. First, as per the co-occurrence criterion, the highest co-occurrences are registered between *stakeholder* and *theory* (4,632), *stakeholder* and *management* (2,910), *management* and *project* (2,638), *stakeholder* and *group* (2,057), *make* and *decision* (1,642) and *stakeholder* and *project* (1,505). As for the significance analysis level, the analysis of the first 10 terms shows the centrality of the business management field in shaping the use of the term *stakeholder* in the selected literature ^13^.

As for the official definitions of the term *stakeholder* used for EU policymaking ( *level 2*) it is possible to quote several here. First, according to the British Standard BS ISO/IEC 38500L:2008 ^14^, a *stakeholder* is « *any individual, group or organisation who may affect, be affected by, or perceive themselves to be affected in a formal perspective by a decision or activity*». On the other hand, IATE underlines that « *stakeholder usually implies some formal relationship to the proposed decision or activity, such as business or property interests that may be affected. 'Interested parties', on the other hand, refers to anyone who might have an interest in the proposed decision or activity, either formally or informally*» ^15^. Finally, the Commission Regulation (EU) No 454/2011 ^16^ defines *stakeholder(s)* as « *any person or organisation with a reasoned interest in train service delivery*», while for the EC Decision 623/2007 ^17^ a *stakeholder* is a « *group that advises the Commission with regard to the Action Programme for Reducing Administrative Burdens in the European Union whose aim is to reduce administrative burdens on businesses arising from EU legislation by 25 % by 2012*».

In conclusion, the three definitions identify respectively three different *stakeholder* characteristics, namely: the capacity to affect/being affected in the first case; the reasoned interest in the second; and the possession of relevant knowledge in the third one.

As for the combination of EU funded research review, academic research semi-systematic review and EU funded projects related to the term *stakeholder* ( *level 3*), it is possible to summarize the results saying that a *stakeholder* might be identified according to interest and influence ( Martinsohn *et al*. 2014), or to the degree/typology of involvement ( Meritxell & Ferraro, 2014). The categorisations proposed by these sources are mostly operational: *stakeholder(s)* can be classified according to their role in the specific field of analysis/action ( Borowiak *et al*., 2012; Kavadas *et al*., 2013; Martinsohn *et al*., 2014; Meritxell & Ferraro, 2014; Svedung & Cojazzi, 2006). Some of them might be identified as key *stakeholders* if considered necessary for the success of a project ( Martinsohn *et al*., 2014). According to the reviewed sources, *stakeholders* should be taken into consideration in order to assess their requirements ( Boden *et al*., 2016; Svedung & Cojazzi, 2006), foster consultation ( Borowiak *et al*., 2012; Matinga *et al*., 2014) and dialogue ( Meritxell & Ferraro, 2014), strengthen their commitment to the issue at stake ( Svedung & Cojazzi, 2006), and finally acquire ( Boden *et al*., 2016; Kavadas *et al*., 2013; Martinsohn *et al*., 2014) and/or transmit ( Svedung & Cojazzi, 2006) relevant knowledge.

Moreover, *stakeholder* theory is used in the attempt of strengthening ethical values (e.g., stronger environmental awareness, gender dimension etc.) as part of a strategic approach to business decision making. All selected papers ( Achterkamp & Vos, 2008; Fassin, 2012; Kaler, 2002; Littau *et al*., 2010; Miles, 2012; Miles, 2017) identify the definition formulated by Freeman (1984) as a milestone in the development of *stakeholder* theories. According to Freeman, a *stakeholder* should be recognised by the fact that she/he/it is capable to affect, or being affected, by the achievement of the organisation’s objectives ^18^.

It must be considered that most of the definitions of the term *stakeholder* taken into account share a characteristic: they take the perspective of a unique subject, usually a firm, and they identify and categorise *stakeholder(s)* according to a specific objective and/or field of action in which the subject is involved. This aspect is particularly highlighted in Miles’ (2012) review as the author collected several answers from different theoretical perspectives on the issue « *Who identifies stakeholders*? »: corporations, enterprises, organisations, management (p. 289). Such a modelling is particularly relevant when dealing with private sector and private sector-like scenarios, where several non-institutional subjects contribute to the definition of a stake.

Regarding the term’s application to EU funded projects, it is possible to remark that a *stakeholder* might be defined by the idea of effect (i.e., it can be impacted, or it can impact by the project) ^19^. In other cases, *stakeholders* are considered as beneficiaries of the project, and they are categorised as *end-users*, potential clients, and decision makers ^20^. Other projects mention « *relevant stakeholders*» ^21^, or talk about « *targeted stakeholders*» ^22^, « *interested stakeholders*» ^23^, or aim at rationalizing *stakeholder*s’ needs and threats ^24^, wants to investigate *stakeholders* through empirical research and expert panels ^25^, or aim at promoting *stakeholder groups* ^26^. So, the approaches can vary a lot from a project to another, and can, thus, impact the use and the perception of what and who *stakeholders* are.

One could draw the conclusion that the majority of the sources under analysis identify *stakeholder*s in terms of a particular stake that is itself established by a key entity, such as a company, institution, or initiative. Then, *stakeholders* are specifically designated as such based on their capacity to effect, be impacted by, or have an impact, as well as their interest in, responsibility for, or claim with regard to the stake. Similar conclusions may be derived from reviews of official definitions, research financed by the EU, academic research, and definitions offered in EU-funded projects.

**3.1.1.2 Methodologies of involvement**

The materials chosen for the EU-funded research part ( *level 1*) primarily consist of scientific contributions, such as chapters from scholarly books or presentations at conferences, in areas like the development of spatial data infrastructure, water management, and energy management. *Stakeholders*’ support is considered as crucial in the development of projects and/or initiatives by all the selected sources. However, they frequently do not specify involvement tools in depth, instead choosing to concentrate on theoretical definitions of *stakeholders’* roles in involvement processes or policy recommendations on the necessity of putting such procedures into practice. ^27^.

As for the analysis of *stakeholders*’ involvement based on academic research ( *level 2*), many sources agree in defining *stakeholders* as both target and active subject of proposed involvement tools ( Becu *et al*., 2003; Dvarioniene *et al*., 2015; Kavadas *et al*., 2013; Ramos *et al*., 2013). Other scholars identify *stakeholders* either as target groups ( Mettepenningen *et al*., 2011) or as active participants ( Vlachokostas *et al*., 2011). According to Tako and Kotiadis (2015) *stakeholders* need to be generally involved in decision-making processes considering the specific knowledge they often bear and to avoid/take into consideration possible conflicting perspectives. Among the tools proposed for the involvement of relevant *stakeholders* it is necessary to mention surveys ( Mettepenningen *et al*., 2011), participatory multi-criteria analysis ( Vlachokostas *et al*., 2011), facilitated dialogue through representation modelling depicting or imitating a selected part of the reality concerning the issue at stake ( Becu *et al*., 2003) or other models ( Ramos *et al*., 2013; Tako & Kotiadis, 2015), e.g., prototypes, also is due mentioning living lab methodology ( Dvarioniene *et al*., 2015), and finally co-creation activities ( Kavaratzis, 2012).

According to the EU funded projects analysis related *stakeholders*’ involvement ( *level 3*) there is a prevalence of collective meetings solutions to involve *stakeholders* – such as focus groups or world café. All inquired projects mentioning *stakeholders*, except for CyberROAD and MEDEA, reported the use of such instruments. Results also show a limited use of training activities (WISER ^28^), quantitative inquiry (TAKEDOWN ^29^, CyberROAD), qualitative inquiry (IECEU), participatory evaluation (BODEGA), and participatory processes (MEDEA) ^30^.

In conclusion, when proposing *stakeholders*’ involvement methodologies – or discussing implemented approaches – the analysed sources take into consideration *stakeholders* as both target groups and active subjects. *Stakeholders*, however, are occasionally treated as either the objects or the subjects of involvement procedures. The above-mentioned sources state that *stakeholders* should be involved to gather pertinent information, avoid and/or manage potential conflict, and ensure the legitimacy and sustainability of the decision-making process. The suggested tools include questionnaires, interviews, focus groups, modeling, multi-criteria analysis, living lab approach, world café, and co-creation activities. They also include structured and/or facilitated discourse.

***3.1.2 Actor(s)***

**3.1.2.1 Meaning of the term**

The major findings of the semantic analysis ( *level 1*) conducted for the keyword *actor* were based on 20 nodes, each of which represented a word that appeared 1,749 times in the chosen literature.

The first element to be noticed is that the lemma *actor* is neither the most quoted – it appears 3,804 times, while *transition* and *power* show respectively 4,481 and 4,876 occurrences – nor the central element, as the strongest co-occurrences are identified between the following terms: *make* and *decision* (1,349), *transition* and *management* (1,060), *power* and *transition* (737), *actor* and *network* (389), *make* and *process* (299), *process* and *decision* (288). This result suggests that, while the literature selected for the term *stakeholder* is more focused on its conceptualisation, literature quoting the term *actor* is more operational. Further lemmas specify the context of action ( *transition, project, use, process, policy, management, decision, plan, change, problem, development*), while others refer to the definition of actor ( *power, social, network, different, group*). At the same time for the significance context the term *actor* emerges as peculiar for the selected literature, as well as terms referring to the theoretical framework in which *actors* are analysed ( *dynamics, sociotechnical, spatial, trajectory*), other lemmas – *housing, renewable, electricity, fossil* – suggesting, again, a more operational profile of the selected literature.

It is significant to highlight that the research based on terms like *actor, multi-actor,* and *multi-actor analysis* did not give relevant results in the IATE Database with regard to the official definitions of the term used for EU policymaking ( *level 2*).

As for the combination of EU funded research review, semi-systematic review of academic research and EU funded projects related to the term *actor* ( *level 3*), it is possible to summarize the results as follows. For the EU funded research review the selected documents belong to two macro-categories: environmental policies and e-inclusion. As it was in the case of the term *stakeholder* (see par. 2.1) they also cover different typologies of documents, e.g., academic papers and policy, survey and technical reports. However, the conducted review provided limited theoretical insights, mostly related to specific fields, such as e-inclusion ( Garrido *et al*., 2012, p. 15) or water management ( Vetere Arellano *et al*., 2007). The selected sources do not provide general definitions of the term *actor*, as these are generally identified according to their specific, contextual role. Perhaps, Garrido *et al*. (2012) propose a categorisation of *actors* according to the sector they belong to: public, private, third sector. In addition, La Notte and Marques (2017) differentiate between two typologies of *actors*: *enabling actors*, who are involved in the production of a specific output, and *beneficiarie*s, which may be intended as *end-users* of that output. Finally, Gordon *et al*. (2013, p. 19) introduce the relevant issue of potential conflict arising among different *actors* according to their different visions of the issues at stake.

For what concerns the Semi-Systematic Review of Academic Research, the selected materials cover different fields of research: spatial and landscape planning, policy and conflict analysis, organisational management, and behavioral simulation, providing a more operational approach, sustained by a smaller body of theory if compared with the term *stakeholder*. Thus, confirming what emerged for the EU funded research review under the same level of analysis. In fact, the boundaries of the term *actor* seem to be blurrier, while sometimes it is proposed as a synonymous of *stakeholder* – explicitly, as in de Bruijn & ten Heuvelhof (2008), or implicitly, as in Kapadia *et al*. (2011) and in Ligtenberg *et al*. (2001).

While the terms *stakeholder* and *actor* often appear to overlap, there are other instances where the use of the term *actor* specifically reveals a network structure that may be more complicated than the one that is defined around the issue at stake in the context of stakeholder theories. What appears to give multi-actor theories their structure represents a process rather than a straightforward stake. This enables a more dynamic picture of *actors*, who are referred to as *players* by Ligtenberg *et al*. (2001), and who exhibit a desire to participate in and engage in interaction rather than just a general interest in the subject at hand ( de Bruijn & ten Heuvelhof, 2008; Ligtenberg *et al*., 2004). Actors can be categorized based on their unique function in the situation being studied ( Ligtenberg *et al*., 2001), or, as proposed by de Bruijn and ten Heuvelhof (2008), taking in account the following characteristics: *stances, interests, resources, relations and repetitive character of the relations*. Avelino and Wittmayer (2016), on the other hand, recommend differentiating between *formal and informal players*, for- *profit and nonprofit actors*, and *public and private actors* when classifying *actors*. Within the parameters of this classification, *actors* may also be separated into groups based on their make-up, including sectors, people, and organizations.

As for the analysis of the use of the term *acto*r in EU funded projects, according to the used sample of H2020 projects it is possible to identify mainly three different approaches. In fact, in the WOSCAP project, *actors* are governmental ones, thus institutions involved in the project field ^31^. On the other hand, according to EUNITY, *actors* are research bodies ^32^, while MEDEA mentions *actors* in the context of the engagement of « *critical mass of security practitioners and actors*» ^33^.

It follows that results regarding the definition of the term *actor* are different from those regarding *stakeholder* since they are conceptually less precise. While the distinction between public, private, and third sector players as well as profit and non-profit actors is frequently made, there is no widely accepted definition of the term *actor* as there was for the term *stakeholder*. It appears that *actors* are grouped and related to one another in a somewhat different way than *stakeholders* since they interact with and/or participate in a process rather than being interested in or impacted by a particular stake. They behave more dynamically than *stakeholders*, therefore they could be classified as participants. *Actors* may participate in more elaborate and articulated connections because they do not relate to a central figure; these structures appear to be more appropriate for elaborate, multileveled institutional contexts. On the other hand, *multi-actor networks* are most appropriate in situations when the legislation or other institutional rules for the specific identification of subjects that need to be involved in participatory procedures. They are probably less operationalized than *stakeholder* structures.

**3.1.2.2 Methodologies of involvement**

The selected materials for the EU funded research part ( *level 1*) consist in academic publications as well as technical reports and similar documents, generally pertaining to the fields of data management and water/waste management. The chosen sources suggest that *actors'* involvement may allow for the exploitation of their knowledge resources ( Paneque Salgado *et al*., 2009, p. 1002) as opposed to their capacity to learn from one another ( Ferraro, 2014), their decision-making capacity ( Craglia & Shanley, 2015), their capacity to act as go-betweens between citizens and political processes ( Garrido *et al*., 2012, p. 12), and them being the selected target of research/project ( Misuraca *et al*., 2014). Other authors affirm explicitly that *actors* should be involved across the whole process ( Ferraro & Martell, 2015). Building on a similar perspective, Paneque Salgado *et al*. (2009) propose the use of participatory multi-criteria analysis to involve actors across the whole processes.

Moving to *actors’* participation according to academic research sources ( *level 2*), the desk-research results consist in methodological or theoretical papers in different fields: quality assessment, cognitive studies, marketing techniques, environmental planning, children engagement and energy policies. Actors are depicted by Krogstrup (1997) as the main characters in a pluralistic setting. According to Jolivet and Heiskanen (2010) and Mäläskä *et al*. (2011), such an environment may be characterized by both rivalry and discourse ( Damart, 2010). The most relevant sources mapping and/proposing tools for enhancing *actors’* participation, are: Krogstrup (1997), proposing User Participation in Quality Assessment (UPQA), Jolivet and Heiskanen (2010), Mäläskä *et al*. (2011) and Cotton and Mahroos-Alsaiari (2015), proposing ex-post evaluation involving *actors*, and Damart (2010), describing cognitive mapping as an instrument supporting the involvement of *actors* in project formulations.

The outcomes of the *actors’* participation based on sources from EU-funded projects ( *level 3*) are quite dismal and reveal a general lack of qualitative questioning, participatory processes, and information and training activities for which *actors’* participation is implemented. In fact, there were only two projects among those reviewed which explicitly foresaw *actors’* participation implemented through participatory meetings (EUNITY) ^34^ and participatory evaluation (WOSCAP) ^35^.

The combination of the sources offers a clear picture of both the major employed tools/methods and the justification for actors' participation. As mentioned in the first part of this paragraph dedicated to *actors*, agents are thought to engage in a pluralistic framework through patterns of both rivalry and cooperation, or discourse. They possess resources, or the capacity for learning, making, and facilitating knowledge. The question of “where” to position *actors'* participation is addressed in a few sources, whether it be throughout the entire process, in the design of a multi-criteria analysis, or at the conclusion of the process, in an ex-post review. Tools that have been suggested or examined range from participated multi-criteria analysis and participated quality assessment to the creation of knowledge centers, group gatherings, and the usage of cognitive mapping.

***3.1.3 End-user(s)***

**3.1.3.1 Meaning of the term**

Moving now on how the term *end-user* is used, 13 nodes representing words with more than 838 (co)occurrences in the chosen literature served as the foundation for the main findings of the semantic analysis ( *level 1*) conducted for this lemma.

The first obvious consideration is that, in the case of *end-user(s),* occurrences were less than for the other two terms (i.e., there were 4,000 occurrences identified for the term *stakeholder*, and 1,749 for the term *actor*). The highest co-occurrence, of course, is that between *end* and *user* (3,044). Other relevant co-occurrences are those between *information* and *systems* (304), *service* and *support* (277), *spreadsheet* and *development* (256), *user* and *use* (215), and *user* and *system* (188). Since the term *end-user(s)* is used so frequently in disciplines like information technology system, most examples occurring in the chosen literature demonstrate that the term *end-user(s)* is more context-specific than the other lemmas under investigation. Regarding the importance context, it demonstrates that the literature that was chosen to support the conceptualization of the *end-user* is mostly concerned with information systems issues.

Considering the *level 2* of analysis (i.e., official definition of *end-user*), according to the ECHA Guidance for downstream users ^36^, an *end-user* is a « *person or body using substances or preparations in an industrial or professional activity (e.g., not a consumer or distributor) who does not supply it further downstream*». On the other hand, Directive 2002/21/EC ^37^ affirms that an *end-user* is an « *ultimate user of a telecommunications service, (i.e., who does not provide public communications networks or publicly available electronic communications services)*». While Regulation (EU) 2019/2020 ^38^ defines an *end-user* as a « *natural person buying or expected to buy a product for purposes which are outside his trade, business, craft or profession*». Eventually, Council document ST 14114/17 ^39^ mentions *end-user* as « *competent authority directly searching CS-SIS, N.SIS or a technical copy thereof*». Therefore, even though they are tailored to distinct settings, all definitions of *end-user* share a common perspective: *end-user(s)* are found at the very end of a series of interactions and actions, such as the end of a production chain. While the third source conflates the concept of *end-user* and *customer*, the last source contends that *end-users* can occasionally include institutional actors in addition to individuals, groups, or for-profit businesses.

Being focused on the combined findings of the semi-systematic review of academic research, the EU funded projects connected to the term *end-user(s)*, and the EU funded research review ( *level 3* of analysis), it is possible to evaluate the following points. Regarding the review of research sponsored by the EU, the materials that were chosen are from several fields, including media analysis, energy policies, fishery management, and security systems. They also cover other document types, including scholarly contributions and technical manuals. The last user of a resource, a tool, or a product is the foundation for all of these definitions of *end-user*. However, no pertinent categorizations are used in the chosen materials. *End-users* are seen as the intended audience for policies and actions ( Bertoldi *et al*., 2013; Punie, 2011; Spisto, 2016), as providers of feedback ( Castro Ribeiro, 2015), as targets for an information effort ( Doyle *et al*., 2016) ^40^.

As for the semi-systematic review of academic research related the use of the lemma *end-user(s)*, the analysed sources cover mostly the fields of IT studies, informatics and entrepreneurial management. Among the reviewed materials, only one paper provides an explicit definition of *end-user(s)*, which, even though related to a specific field of inquiry (IT studies) may be easily extended to other contexts too: « *An end-user is any organizational unit or person who has an interaction with the computer-based information system as a consumer or producer/consumer of information*» ( Cotterman & Kumar, 1989, p. 1315). On the other hand, Scherrer-Rathje and Boyle (2012) suggest that the identification of *end-user(s)* as such depends upon the context of analysis. For instance, they define as *end-users* (of entrepreneurial strategic choices) several categories of employees and managers.

Therefore, it is possible to argue that the last, ultimate user of a good, tool, resource, or service is the recurring concept attached to the word *end-user(s)* through the many methodological approaches. Nevertheless, this does not imply that an *end-user* is always a consumer—it could be, for instance, a competent authority using a particular piece of software or a technical user of a particular tool, device, or service—nor that it is situated at the very end of a fictitious chain of actors. For instance, an intermediate developer deploying a particular instrument might be an *end-user* of that instrument. In other words, the fundamental factor directing the entire structure is the rigid link between *end-users* and the objects of their usage.

**3.1.3.2 Methodologies of involvement**

In terms of the analysis of *end-user* participation, EU-funded research sources ( *level 1*) continue to be the starting point. The chosen materials cover the management of fisheries, soil studies, and spatial information and include scholarly papers as well as technical reports and related materials. The proposed *end-user* participation typology is where the selected references diverge most. Most of the sources advocate for the creation of feedback mechanisms ( Abella *et al*., 2013; Castro Ribeiro & Guillen, 2016; Hengl & Husnjak, 2006). Ben-Dor *et al*. (2008), on the other hand, advocate constant communication between developers and *end-users* across all stages of a project.

On the other side, when the focus is turned to *end-users’* engagement according to academic research ( *level 2*) the results indicate a preponderance of methodological papers as well as case studies reviews. According to L’Astorina *et al*. (2015), *end-users* are a product’s final users, with their definition overlapping with *stakeholders*, Almirall *et al*. (2012) contend that *end-users* are co-creators as opposed to being merely a topic of research. The length of time suggested for *end-users’* participation in sources can be categorized, as was already the case with *actors*’ participation. Some authors back their participation throughout the entire process ( Almirall *et al*., 2012; L’Astorina *et al*., 2015; Othman, 2007; Sun, 2013).

Finally, the findings based on the sources employed for the analysis of *end-user* engagement in EU financed projects ( *level 3*) reveal a general lack of quantitative research, participatory processes, and participatory assessment for which *end-user* involvement is implemented. Interviews and group meetings were used to see how the IECEU ^41^ project approached its *end-users*, as stated in its dissemination plan. BODEGA ^42^ offers an online platform and coordinated a meeting with a scenario-focused agenda. In conclusion, there have been few discoveries in the sources regarding the justification for *end-user(s)* involvement. From a methodological perspective, feedback mechanisms seem to be the most often used instruments for user involvement, primarily including *end-users* in monitoring and evaluation activities. On the other hand, some sources advocate including *end-users* throughout the entire process, promoting communication between *end-users* and developers at every stage of a project.

### 3.2 The context of action of the ARESIBO Participatory Model (APM)

In order to frame the context of action of the APM, it is necessary to focus the attention on the key concept of border first. For the purpose of this paper, the border should not be interpreted only as a geographical locus, but rather as a complex context of interaction among different *stakeholders*, *actors* and *end-users*. According to some scholars, borders work as sites « *at and through which socio-spatial differences are communicated*» ( Van Houtum, 2005, p. 672) and thus the construction of the border may be understood as the result of bordering practices, discursive and emotional as well as technical ( Kinnvall & Svensson, 2014). Building a border entails the definition of a mobile equilibrium between surveillance techniques and procedures, respecting individual privacy, and trying to provide security. Borders lose their typically associated “fixed” and “static” image when understood as contexts of interaction. The extension of borders’ geographic locations, often known as the “diffuse” border ( Pavone & Degli Esposti, 2012, p. 559), and the externalisation of EU frontiers, become simpler to recognize ( Guild *et al*., 2008). More specifically, in recent years, there has been a strong security link between irregular forms of human mobility and security, both in terms of what is known and what policies are in place ( Carrera & Guild, 2007). At the same time, border control concerns have been more closely associated with anti-terrorism objectives as a result of the strong but contentious correlation between immigration and anti-terrorism strategies ( Argomaniz *et al*., 2015).

Building on this complex and multifaceted idea of border, and on the centrality of the security-privacy dichotomy, it is now crucial to introduce three related fundamental concepts from a literature review perspective. These concepts are *surveillance*, *security*, and *privacy*.

From an historical standpoint, Dratwa affirms that the term surveillance comes from « *the French verb surveiller […], from sur- ‘over’ and veiller ‘to watch’, from Latin vigilare, from vigil ‘watchful’. Interestingly, ‘surveiller’ carried with it from the start a tension between the meanings of watching over, of taking care of, and of suspicion and control. It also comprised from the start the complementary notion of watching over oneself and one’s own behaviour*» (2017, XIX). Moving on to a more specific topic linked to the focus of this study, surveillance may be defined as the collection of tools and practices used to compromise privacy in order to ensure security. The definition of surveillance is, of course, inextricably linked to those of privacy and security. Therefore, the term surveillance has no absolute value *per se*, but its definition is indeed the result of social, political, and cultural processes of legitimization and delegitimization.

On the other hand, the literature review shows that security is a very broad, ambiguous, and mobile term. From a theoretical point of view, it could stand for all the actions – usually institutional – claiming to reach a positive output in different contexts ( Wæver, 1993). As a result of this, Friedewald *et al*. (2015, p. 42) felt an effort of specification was needed, thus identifying seven typologies of security: physical security, political security, socio-economic security, cultural security, environmental security, radical uncertainty security, information security. The suggested list leaves room for additional definitions of "security" that, depending on the precise context of use, may have a positive or negative connotation ^43^. As a last observation, it is important to draw attention to Guild, Carrera, and Balzacq’s claim that security, as opposed to privacy,« *is not a value as such*» (2008, p. 9).

Turning now to the privacy aspect, Gavison (1980, p. 423) offers a general definition of privacy as follows: « *the extent to which we are known to others, the extent to which others have physical access to us, and the extent to which we are the subject of others' attention*». The concept of privacy varies across space and time, being continuously shaped by socio-economic, political, cultural, and technological factors. Changes in technology, more particularly, « *have continually required a more precise re-working of the definition in order to capture the ethical and legal issues that current and emerging surveillance and security technologies engender*» ( Friedewald *et al*., 2015, 41). Such instability led to the point reported by Solove, when he states: « *Privacy is a concept in disarray. Nobody can articulate what it means*» ( Solove, 2006, p. 477).

More recent definitions of the term, however, show that the focus still lays on the issue of availability of sensitive information – not necessarily data in the strict sense of the term – pertaining to individuals. Riley (2007) defines privacy as the right to have one’s personal information safeguarded against access by the government and/or commercial organizations interested in doing so for purposes of trade, profit, or other reasons that go beyond the exceptions allowed by law. Such definition introduces the aspect of exceptional circumstances which, according to this perspective, limit the exercise of the right to privacy. ^44^.

Also, regarding the goal of this essay, it is crucial to move away from an abstract conception of *security* – and from *surveillance* as the collection of tools that ensure it – to *securitization*, a procedure developed from a socially constructed conception of security. As Léonard emphasized, « *what security scholars can and should study is the process through which an issue becomes socially constructed and recognised as a security threat*» ( Léonard, 2010, p. 235). It follows that securitisation policies and practices address issues that have been ‘securitised’ (i.e., constructed as such through securitizing discourses). What is meant as discourse vary from author to author: some scholars consider discourses mainly as speech acts ( Wæver, 1993), while others consider them as performative practices in the broader sense, ranging from administrative tools to the deployment of technical knowledge ( Bigo, 2000). Léonard (2010) remarks that, in the EU context, securitisation processes have been mainly enacted through practices ^45^.

As a result of this approach, securisation procedures are becoming more and more characterized by the employment of new technologies, or SOSTs. Two outstanding examples of this include the application of drone technology and the use of biometrics in border management and control. Implementing SOSTs frequently necessitates defining specific infrastructures for data sharing and storage, which introduces significant privacy and data protection problems ( Pavone & Degli Esposti, 2012) ^46^. In a historical context in which technology could have bridged the gap between institutions and citizens, SOSTs development appears to be caught in what has been dubbed a proximity paradox ( Lodge, 2005): the growing proximity between institutions and citizens raises concerns and suspicions more than trust and legitimacy.

The relationship existing among security and privacy, often conceived as a balance, appears thus now more complex than ever before. As underlined by Pavone and Degli Esposti, SOSTs engage citizens in the controversial process of trading « *part of their privacy in exchange for enhanced security*» (2012, p. 558) and even to take on themselves the burden of proof ( Argomaniz *et al*., 2015, p. 200). Similar dynamics are especially ambiguous because it is challenging to measure how well these technologies promote security while preserving fundamental rights and the rule of law ( Argomaniz *et al*., 2015; Guild *et al*., 2008). Last but not least, Dratwa (2017) characterizes the current stage of SOSTs growth as “beta”, and as a result, it is characterized by ambivalent processes that both exploit and strengthen citizens’ rights.

Considering all these points, as well as the literature review provided in previous sections of this paper, and the existent, reliable and consolidated frameworks for analysis and implementation of engagement/involvement activities elaborated by Ostrom and by ISIG/Council of Europe (i.e., the Institutional framework of Common-pool resources management – Ostrom, Gardner and Walker, 1994, and the Civil participation framework of the Council of Europe – ISIG/CoE 2017) it is possible to introduce in the next paragraph the three components of the ARESIBO Participatory Model (i.e. the ARESIBO Participation Framework - APF, the ARESIBO Participation Strategy - APS, the ARESIBO Participation Action Plan - APA).

### 3.3 The ARESIBO Participation Framework (APF), the ARESIBO Participation Strategy (APS) and the ARESIBO Participation Action plan (APA)

As a general preliminary note to the overall presentation of the APM’s components and phases, it must be highlighted here that the methodological framework proposed by Ostrom *et al*., (1994), together with the approach and the tools elaborated by the Council of Europe and by ISIG in the framework of the Civil participation framework ( ISIG/CoE, 2017), represent the solid basis on which the model has been built and tested. Both frameworks guarantee the possibility to resort to an innovative and reliable tool in order to elaborate a focused analysis and consistent implementation of involvement activities.

***3.3.1 ARESIBO Participation Framework (APF)***

The *ARESIBO Participation Framework (APF)* is the APM methodological element. It consists of a heuristic framework that specifies the involvement’s goals and techniques. In the cited Institutional framework of Common-pool resources management ( Ostrom *et al*., 1994), the characteristics of the physical world, the characteristics of the community, and the rules-in-use all contribute to shaping an action arena, which includes action situations (i.e., the opportunity for *actors* to interact, exchange resources, knowledge, etc.) and the *actors* themselves. The action arena generates some interaction patterns that lead to a result. The result is then considered as the subject of evaluation criteria. For the purpose of APF, the Action arena is understood as the SOSTs development process. In order to accomplish this, the aforementioned framework was interpreted in order to elaborate the APF. Attributes of the physical world are intended within the APF as the Border Area under focus for the purpose of designing and implementing involvement activities. Geographical and infrastructure features that define the border region at issue serve as a metaphor for the “real world” in this sense. The project demo-sites and pilots provide the border areas that are being analyzed for ARESIBO. The APF defines rules-in-use as the collection of border security policies and procedures that are in effect at a particular border. All these factors combine to form the APF’s Action Arena (i.e., the SOSTs development process), which is where action circumstances and *actors* for upcoming engagement activities were found.

The *Actors* are envisaged within the APF to be the targets of the involvement actions in the future. The analysis’ findings, which are presented in paragraph 3.1, demonstrate how the definitions of the targets of involvement – *stakeholders*, *actors*, and *end-users* – tend to overlap. In particular, the definitions of *actors* and *end-users* use the term *stakeholder* as a point of comparison.

Using the aforementioned definitions as a foundation, the APF suggests a taxonomy for further target identification that takes into account *impact* and *acceptance*, two types of dimensions that ultimately describe the relationship between the future target, the SOSTs development process and related end-results/product. It will be feasible to emphasize the potential relevance of targets in the involvement process, particularly in terms of the kinds of information, feedback, and insights that will need to be gathered, by analysing targets based on these aspects.

The first dimension, the *impact* of the SOST development process (i.e., What type of impact will the final results of the SOSTs development have on the target?), can be a *direct impact* (i.e., the target has a direct relation with the products/end-results of SOST development process, such as direct deployment of the product or direct exposure to the product) or an *indirect impact* (i.e., the target has an indirect relation with the products/end-results of SOST development process). The second dimension is the *acceptance* of the SOST development (i.e., What type of acceptance is expected from the target in relation to the results of the SOST development process? What kind of feedback is thus envisaged from the target?) and it is articulated in two sub-dimensions as well: *technical acceptance* (i.e., the target expresses ultimately its levels of acceptance with the deployment of the product, thus with the usability of the product) or *societal acceptance* (i.e., the target expresses ultimately its levels of acceptance with the perceived effects of the deployment of the product in a specific (societal) context).

Ultimately, the Taxonomy for Targets identification, allows for depicting four main profiles of Targets of involvement: *Citizens, Stakeholders, Actors and End-users*.

Moving on to the identification of *Action Situations* for the ARESIBO Participation Model, the APF suggests a Taxonomy that considers two categories of dimensions, namely *Capacity and Interest*, which ultimately describe the potential interaction of the identified target and the SOST development process itself. *Capacity* is defined as the target's type and level of knowledge in relation to the SOST development process. In this respect, capacity is defined in terms of two dimensions: *context capacity*, which denotes a high degree of awareness and understanding of the particular (societal) context at issue, and *technical capacity*, which denotes a high or expert level of technical knowledge of the SOST. On the other hand, *Interest* is defined as the degree of the targets’ readiness to interact for the growth of the SOST. In this sense, interest can be divided into two sub-dimensions: *direct interest*, which refers to targets who are highly willing to participate in or provide feedback on the development process, and *indirect interest*, which refers to targets who are less willing to participate in or provide feedback on the development process. The four primary patterns of interaction that can be represented using the Taxonomy of Action Situations are as follows: *Information, Advice, Discussion, and Partnership.*

In the end, the APF permits the identification of 8 dimensions that characterize the framework for involvement and participation in the SOST development process, as follows: *Direct impact, Technical acceptance, Technical capacity, Indirect interest, Indirect impact, Societal acceptance, Context capacity, Direct interest*. The 8 dimensions are deployed in the APF with a twofold objective. First, performing an initial context analysis (i.e., SWOT analysis) that will shape the Action Plan for a Participation oriented SOST development. Second, is due performing monitoring and evaluation activities (i.e., evaluative criteria) on the implementation of the Participation oriented SOST development (i.e., involvement activities).

***3.3.2 ARESIBO Participation Strategy (APS)***

As previously mentioned, the APM seeks to ensure a consistent and uniform framework within which various pertinent targets (i.e., *citizens* and *communities*, *stakeholders*, *actors*, and *end-users*) are effectively and efficiently involved in the development of ARESIBO technologies throughout the project cycle.

According to this perspective, the *ARESIBO Participation Strategy (APS)* comes after the APF and determines the context and targets. It seeks to establish the goals and particular objectives of engagement for certain activities (such as pilots) and situations (e.g., ARESIBO pilot communities). The APS aims at ensuring that all ARESIBO products are created in such a way that they are relevant to and beneficial to *stakeholders* and people in a particular context, as well as valuable to and owned by *end-users* and *actors*.

To accomplish this, the APS designates the following points as the overall objectives of all ARESIBO engagement activities. First, in accordance with the categories envisioned by the APF ( *citizens, stakeholders, actors*, and *end-users*), each ARESIBO pilot site’s targets of involvement must be clearly and consistently mapped, both internally and externally Second, it is necessary to involve both internal and external targets in order to collect feedback on the usability, applicability, and usefulness of ARESIBO tools (from *actors* and *end-users*), as well as on the tools' overall perceived impact and degree of societal acceptance (from *stakeholders* and citizens). Third, it is essential to have the assurance that all project partners have the capabilities and tools necessary to participate in the engagement process.

The APS, to be intended as an engagement strategy, promotes a co-creation approach in the SOSTs development process, thus fostering a structured engagement of targets in different phases of the project. The strategy is divided into two key stages. The first one is *co-design*, which refers to engagement activities meant to identify and analyse issues and potential solutions. These activities take place during the early stages of the development process and have the objective of determining the precise needs and requirements of the target audience. The second is *co-production*, which refers to engagement activities that are focused on implementing or testing the suggested solutions. These activities refer to the implementation phase.

Moreover, the involvement activities were designed so to reflect the following core values: *Participation, Responsiveness, Efficiency & effectiveness, Openness & transparency, Innovation, Diversity, Accountability.* Finally, it is necessary to recall that in order to sustain the involvement activities, attention should be also paid to viable incentives for participation ^47^.

***3.3.3 ARESIBO Participation Plan (APA)***

The *ARESIBO Participation Action Plan (APA)* represents the operationalisation in concrete activities of the overall APM. The APA is a living document that offers the operational procedures and tools that partners in the involvement process must use, as well as the instruments for the process’ monitoring and assessment. 

The APA establishes the responsibilities (i.e., ownership of involvement activities), objectives (i.e., what is the scope of the involvement activity at hand, such as co-design or coproduction, and who are the targets, such as *citizens, stakeholders, actors*, or *end-users*), outputs (i.e., desired concrete results of the involvement activities, such as type of data or feedback collected from targets), and timeframe for each involvement activity (i.e., setting the involvement activities in the overall project GANTT). Moreover, to ensure an efficient implementation of the involvement activities, monitoring and evaluation actions are envisaged by the APA – such actions are embedded/engrained within the monitoring and evaluation mechanism of the overall project.

Stemming from the literature review and related analysis around the methods and tools deployed in involvement activities, as well as from the project map of involvement activities, the following activities have been identified by the APA as relevant for the purpose of the ARESIBO Participatory Model: *targets mapping, questionnaires, workshops*.

The major goal of the targets mapping is to identify the groups that should be included in each of the ARESIBO trial sites in accordance with the proposed taxonomy included in the APM, including citizens, *stakeholders*, *actors*, and *end-users* both inside and outside the project consortium. By basing the questionnaires’ content on particular SOSTs development steps, their primary goal is to collect input from specified targets at various stages of SOSTs development and on particular issues. Workshops are meant to be interactive gatherings that focus on certain SOSTs development steps and aim to collect feedback from *stakeholders* and citizens at various stages of SOSTs development and on particular subjects (e.g., impact of SOSTs, societal acceptance, etc.).

## 4 Conclusions

At this stage, the APM is (still), rather than a consolidated theoretical model, a working hypothesis, built on the methodological approach introduced by this paper (i.e. scientific literature, semantic analysis and EU funded research review), combined with the tools/methods for engagement proposed by Ostrom *et al*., (1994) and CoE/ISIG in their Civil Participation Framework (2017) and resulting in an original heuristic/methodological framework. Despite its consolidated scientific and theoretical basis, as well as the relevant results acquired thanks to its partial implementation in the framework of ARESIBO, it would benefit from further development, though analysis, application and testing. Its application has been indeed limited to aspects related with the analysis of potential technical and ethical challenges in the SOSTs field, partially deriving from the level of distrust that HRGs, CSOs, but also citizens in general, seem to express towards surveillance technologies. In other words, the model served as the basis for the design of activities targeting HRGs, CSOs, and local authorities, to explore societal and technical acceptance of SOSTs and to question about the existence of a “common ground” for future improvements through information, consultation, dialogue and partnership approaches.

The high degree of adaptation of the methodological framework, however, would allow for its future operationalisation in several other contexts. In particular, the methodological framework on which the APM is built could be particularly useful for European projects and initiatives dealing with socially sensitive issues that call for the participation and co-design effort from a variety of different *actors, stakeholders,* and *end-users*, such as border management agencies. The peer review process could surely contribute to enhance its flexibility and usability. 

Additionally, through such framework, future project consortiums could have access to a wide range of instruments to foster engagement, thanks to the high level of interoperability of the tools associated with it (such as surveys, workshops, and interviews). In this perspective, the model might be adopted more broadly at the EU level and/or by border agencies (such as FRONTEX) as a good practice to encourage the inclusion of HRGs and CSOs cases to reduce the perception of marginalization of such actors and foster a “common ground” based on knowledge, mutual trust, and cooperation.

## Ethics and consent

Ethical approval and consent were not required.

## Data Availability

No data are associated with this article.

## Footnotes

^1^ UN, Office of the High Commissioner for Human Rights (OHCHR), 2014. *Recommended Principles and on Human Rights at International Borders.* See https://www.ohchr.org/Documents/Issues/Migration/OHCHR_Recommended_Principles_Guidelines.pdf.

^2^. Among others see: https://www.unhcr.org/4963237c11.pdf. See also https://www.iom.int/sites/g/files/tmzbdl486/files/defaul/pp_cross-border_human_mobility_amid_and_after_covid-19_policy.pdf.

^3^; See https://frontex.europa.eu/accountability/fundamental-rights/consultative-forum/general/.

^4^ The European Integrated Border Management can be defined like the « *National and international coordination and cooperation among all relevant authorities and agencies involved in border security and trade facilitation to establish effective, efficient and coordinated border management at the **external EU borders**, in order to reach the objective of open, but well controlled and secure border*» ( https://ec.europa.eu/home-affairs/pages/glossary/european-integrated-border-management_en). The legal basis is given by Art. 4 of Regulation (EU) No 2016/1624 (European Border and Coast Guard Regulation). A fundamental part of this approach is represented by FRONTEX, that promotes, coordinates and develops European border management in line with the EU fundamental rights charter and the concept of Integrated Border Management. The agency monitors and analyses the situation at the EU external borders. It carries out vulnerability assessments to evaluate the capacity and readiness of each Member State to face challenges at its external borders. It assists EU countries in training national border guards and develops common training standards. It also participates in the development of research and innovation related to the control of external borders, and it assists EU countries in circumstances requiring increased technical and operational help at external borders which may imply support - in cooperation with other agencies like Europol and Eurojust– to detect and prevent organised cross-border crime and terrorism.

^5^ The general objective of the ARESIBO project is: « *[…] improving the efficiency of the border surveillance systems by providing the operational teams and the tactical command and control level with an accurate and comprehensive information. The pillars of research in ARESIBO were three-fold: 1. Set-up a complete configuration at tactical and execution level to optimise the collaboration between human and sensors (fixed and mobile), 2. Improve situation awareness by enhancing the understanding of the situation through adapted processing of sensor data, correlation between heterogeneous data and information and creation of knowledge through deep learning techniques and 3. Create a situation awareness capability at C2 level that will combine reports on previous missions, real time situation understanding and threat analysis for future actions.*», in https://www.aresibo.eu/content/objectives.

^6^ The same analysis has not been performed for the ‘citizens’ category. According to the existing literature and its operational uses, ‘citizen’ has been broadly understood, for the purpose of the ARESIBO involvement activities, as the entirety of residents of a specific border area and as the overall European Union’s citizens.

^7^ The first 7 results according to Google Scholar relevance rank have been selected and, when available, inquired in the light of the abovementioned research questions. So, 18 sources in total were analysed in the semi-systematic review of academic research (7 stakeholder definition, 7 multi-actor definition, 4 end-users taxonomy).

^8^ 230 sources were analysed for the term *stakeholders*, 78 sources for the term *actors* and 28 sources for *end-users*.

^9^ Sources have been analysed using Orange software. The analysis is based on: the "co-occurrence network", that is a diagram showing the most frequent interconnections between the inquired terms and other lemmas used in the selected literature; and the "significance analysis", which explores the literature related to one of the analysed terms comparing it with the rest of the whole literature to show the lemmas that specifically characterise that analysed term.

^10^ According to its official presentation, « *IATE (Interactive Terminology for Europe) is the EU's terminology database. It has been used in the EU institutions and agencies since summer 2004 for the collection, dissemination and management of EU-specific terminology*». See https://iate.europa.eu/home.

^11^ The JRC is the European Commission's science and knowledge service advising and supporting the development of EU policies. Results have been selected according to JRC Publications Repository relevance ranking system and only online available documents have been examined. See https://joint-research-centre.ec.europa.eu/index_en.

^12^ According to its official presentation « *The Community Research and Development Information Service (CORDIS) is the European Commission's primary source of results from the projects funded by the EU's framework programmes for research and innovation (FP1 to Horizon 2020*)». See https://cordis.europa.eu/about/en.

^13^ Such centrality can be clearly ascertained by the frequent use of sub-definitions of the term *stakeholder* pertaining to the business management field (i.e., *shareholder* and *stockholder*); as well as the frequent references to business approaches and frameworks (i.e., *ethic, ethical, capitalism, corporation*).

^14^ https://www.iso.org/obp/ui/#iso:std:iso-iec:38500:ed-1:v1:e.

^15^ https://iate.europa.eu/entry/result/900023.

^16^ Commission Regulation (EU) No 454/2011 of 5 May 2011 on the “ *Technical specification for interoperability relating to the subsystem ‘telematics applications for passenger services’ of the trans-European rail system*”, https://eur-lex.europa.eu/legalcontent/EN/TXT/?uri=uriserv%3AOJ.L_.2011.123.01.0011.01.ENG.

^17^ 2007/623/EC: *Commission Decision of 31 August 2007 setting up the High-Level Group of Independent Stakeholders on Administrative Burdens*. See https://eur-lex.europa.eu/legalcontent/en/TXT/?uri=CELEX%3A32007D0623%2801%29

^18^ Several sources highlighted as relevant another categorisation proposed by Mitchell *et al*., (1997) which measure the *stakeholders*’ salience according to three criteria: the power a *stakeholde*r may exercise on a firm; the legitimacy of a *stakeholder* in relationship to a firm; the urgency of a *stakeholder’s* claim vis-à-vis a firm. Fassin (2012) further specifies the characteristics of legitimacy bounds by proposing a categorisation of *stakeholders* as follows. The first category is represented by constituents who have a real stake in the company, or *stakeowners*. The second are pressure groups that may influence the firm, or *stakewatchers*. The third category are regulatory institutions who exercise control and regulation functions, or *stakekeepers*. And finally, as fourth category there are groups which aim to have a voice in decision-making processes, or *stakeseekers*.

^19^ IECEU Improving the effectiveness of capabilities in EU Conflict Prevention (see https://ieceu-project.com/).

^20^ RED Alert - Real-time Early Detection and Alert System for Online Terrorist Content based on Natural Language Processing, Social Network Analysis, Artificial Intelligence and Complex Event Processing Please see https://cordis.europa.eu/project/id/740688/it.

^21^ EUNITY Cybersecurity and Privacy Dialogue between Europe and Japan (see https://www.eunity-project.eu/).

^22^ WISER Water Bodies in Europe: Integrative Systems to Assess Ecological Status and Recovery ( http://www.wiser.eu/).

^23^ MEDEA The Mediterranean & Black Sea Security Practitioners’ Network ( https://www.medea-project.eu/).

^24^ CyberROAD Development of the Cybercrime and Cyberterrorism Research Roadmap ( https://www.cyberroad-project.eu/).

^25^ Takedown (Identify, Prevent, Respond) - Developing effective and efficient security solutions against organised crime and terrorist networks ( https://www.takedownproject.eu/overview/), and BODEGA Proactive Enhancement of Human Performance in Border Control ( https://bodega-project.eu/).

^26^ DOGANA Advanced Social Engineering and Vulnerability Assessment Framework ( https://www.dogana-project.eu/).

^27^ Perhaps, when a lower level of participation is proposed, *stakeholders* are seen mainly as a target to be inquired ( Matinga *et al*., 2014). On the other hand, when the participation level increases, *stakeholders* may form communities of interest ( Craglia & Annoni, 2007) and/or engage in dialogues with expert and policy makers ( Grizzetti *et al*., 2010b). Furthermore, they can also be the protagonists of participatory modelling processes ( Grizzetti *et al*., 2010a). Generally, *stakeholders*’ involvement is recognised by these sources as key in guaranteeing sustainability and legitimacy in a decision-making/implementation process ( Craglia & Annoni, 2007; Grizzetti *et al*., 2010a). *Stakeholders* are also often framed as producers of relevant, local knowledge ( Grizzetti *et al*., 2010a) which may positively affect both content and process.

^28^ Communication & Stakeholder Plan, pp. 10-13 https://www.cyberwiser.eu/system/files/CYBERWISER.eu_D6.4_Communication_Stakeholder_v0.12%281%29.pdf.

^29^ https://www.takedownproject.eu/overview/.

^30^ See footnotes n. 17, 18, 20, 21 for all details related quoted projects.

^31^ See https://www.woscap.eu/deliverables/index.html.

^32^ See https://www.eunity-project.eu/en/.

^33^ See https://www.medea-project.eu/.

^34^ See footnote n. 21.

^35^ See footnote n. 22.

^36^ https://echa.europa.eu/documents/10162/23036412/du_en.pdf.

^37^ Directive 2002/21/EC of the European Parliament and of the Council of 7 March 2002 on *a Common regulatory framework for electronic communications networks and services (Framework Directive),* https://eur-lex.europa.eu/legal-content/EN/TXT/?uri=CELEX%3A32002L0021.

^38^ Regulation (EU) 2019/2020 *Laying down eco-design requirements for light sources and separate control gears pursuant to Directive 2009/125/EC of the European Parliament and of the Council*, https://eur-lex.europa.eu/legal-content/EN/TXT/?uri=uriserv:OJ.L_.2019.315.01.0209.01.ENG.

^39^ Council document ST 14114/17: *Proposal for a Regulation of the European Parliament and of the Council on the use of the Schengen Information System for the return of illegally staying third-country nationals*, https://eur-lex.europa.eu/legal-content/EN/TXT/?uri=celex%3A52016PC088.

^40^ « *Those addressing security issues as opposed to adopting video analytics for operational purposes, such as shopper footfall analysis*» (p. 7).

^41^ Dissemination Plan, p. 14. See https://www.ieceu-project.com/wpcontent/uploads/2016/12/D8.1-Dissemination-Plan-PU.pd.

^42^ See footnote n. 23.

^43^ The formulation “social security”, for instance, tend to bear a positive meaning in nowadays European societies, while an expression such as “coercive security” is a much more contested field. Pavone and Degli Esposti summarize security as « *the right and duty of national governments to ensure citizens’ personal safety*» (2012, p. 558). In this case, they argue, security is presented as « *freedom from fear*» as in Manners (2006, p. 192) or « *human security*» ( Liotta & Owen, 2006, p. 40).

^44^ Finn *et al*. (2013) identify seven typologies of privacy to further address the ambiguity of the concept's definition: privacy of the person, privacy of behavior and action, privacy of communication, privacy of data and image, privacy of thoughts and feelings, privacy of location and space, and privacy of association (including group privacy).

^45^ When it comes to the utterance of speech acts that secure national interests, the EU, in fact, does not have the same authority as national, sovereign States. At the same time, the intricate institutional framework of the EU, with its extensive web of laws and other governmental instruments, promotes sound securitization practices. Following a similar path, when discussing the issue of securitisation, Kinnvall and Svensson define it as a “pragmatic act”, consisting of: «( *i) a relatively stable system of heuristic artefacts or resources (metaphors, image repertoires, stereotypes, emotions), (ii) discursively mobilized by an agent, who (iii) works persuasively to prompt a target audience to build a coherent network of implications (feelings, sensations, thoughts, intuitions) that concurs with the enunciator’s reasons for choices and actions, by (iv) investing the referent subject with such an aura of unprecedented threatening complexion that (v) a customized political act must be undertaken immediately to block its development within a specific spacetime continuum or a social field*» (2014, 2). Such logic is clearly at play when migration policies are framed as a response to a security threat ( Kinnvall & Svensson, 2014; Léonard, 2010), in policy fields such as EU external borders management.

^46^ This is as thoroughly analysed in Marin (2017), concerning the use of drone technology in border surveillance.

^47^ In the field of research design an incentive is usually proposed as a strategy to increase recruitment and retention ( Bower *et al*., 2014); more generally, an incentive operates to overcome barriers which prevent relevant/interested actors from participating in a research process, and to enhance positive side effects resulting from the participation. Barriers are those linked with time, effort, or financial costs, as well as discomfort in relation with research procedures. Taking the perspective of economic theory, as proposed in Parkinson *et al*. (2019), an incentive can be framed as a contract between a principal and multiple agents. The literature often warns about the side effects – being positive and/or negative, thus unintended – of incentives on the research process. Altruistic benefits and recognition as key actors in the research process may be taken into consideration as positive side effects embedded in the research procedures: such positive effects may be labelled as "implicit" incentives, when they are not explicitly stated as such ( Parkinson *et al*., 2019). Incentives may produce also unintended, negative consequences, for instance benefiting specific groups of *stakeholders* more than others. Incentives may be grouped in two macro-areas: monetary, non-monetary incentives ( Parkinson *et al*., 2019).
