# Supplementary material for: Towards the engagement of citizens in SOSTs decision-making: participatory models setting a common ground for border surveillance and respect of fundamental rights. Findings from ARESIBO H2020 project
Source: Open Res Eur. 2023 Jan 25;3:15. [Version 1] doi: 10.12688/openreseurope.15079.1 (PMC11358682; doi:10.12688/openreseurope.15079.1)
Supplement: Peer review history

# Reviewer report: Reviewer response for version 1

**DOI:** 10.21956/openreseurope.16307.r30717

**Published:** 2024-08-28

**Authors:** Jernej Letnar Černič

**Affiliations:** 1 Faculty of Government and European Studies, Nova Univerza in Slovenia, Kranj, Slovenia

This article comprehensively discusses and analyzes participatory models establishing minimum common denominators to respect human rights and fundamental freedoms during border surveillance. The report is methodologically sound research. The authors discuss and adapt ARESIBO Participatory Model (APM) to protect human rights during border surveillance. They provide suggestions on how to implement APM in practice. The report is a welcome contribution to enhance participation and human rights protection during border surveillance.

Are sufficient details provided to allow replication of the method development and its use by others?

Yes

Is the description of the method technically sound?

Yes

Are the conclusions about the method and its performance adequately supported by the findings presented in the article?

Yes

Is the rationale for developing the new method (or application) clearly explained

Yes

If any results are presented, are all the source data underlying the results available to ensure full reproducibility?

Yes

Reviewer Expertise:

Human rights law, rule of law, constitutional law, research methodology

I confirm that I have read this submission and believe that I have an appropriate level of expertise to confirm that it is of an acceptable scientific standard.

# Reviewer report: Reviewer response for version 1

**DOI:** 10.21956/openreseurope.16307.r33098

**Published:** 2023-07-18

**Authors:** Andres De Castro García

**Affiliations:** 1 Universidad Nacional de Educacion a Distancia, Madrid, Community of Madrid, Spain

Human Rights and Border Management are not "strictly interlaced".

 It is easy to say that CSOs and HRGs need to participate in the decision making, but it is important to say how. Often they have contradicting interests. Which ones should prevail? 

 From the academic perspective, it is simple to state that "it is not relevant to consider borders only as linear and static expressions of state power" but it is actually very relevant. One Academia and practitioners are so far from each other the chances of cooperation and the possibility of looking for the truth are low. 

 The paper does not clarify the incompatibility of the legitimate hopes of the migrants/refugees/asylum seekers (arrive to Europe) and the legitimate national interest of many European Union members (control their borders). Thus, "common ground" "mutual trust" and "cooperation" is not possible.

 I wonder, very respectfully, how can we integrate the work of FRONTEX and of HRGs and CSOs and Academia. If I am a member of an NGO and I believe in open borders and total freedom of movement (very legit) I might want to block the work of FRONTEX or of a given police force trying to return the migrants. If I work for FRONTEX or a police force, I can't see the role of those organisations as a good idea. If I am an Academic, the role would be (as I understand it) to explain the real situation from a realist perspective more than trying to accommodate both attitudes and ideas into one. Which is not -in my humble opinion- possible. 

 When approved with reservations, I just want to pose the following question: Don't you think it would be more useful to use realism as the way to deal with the issue? Don't you think it would be a better way to "solve" the problem in hand? 

 I am glad that I got to have the option to address this issue and count on me if there is anything needed.

Are sufficient details provided to allow replication of the method development and its use by others?

Partly

Is the description of the method technically sound?

Partly

Are the conclusions about the method and its performance adequately supported by the findings presented in the article?

Partly

Is the rationale for developing the new method (or application) clearly explained

Yes

If any results are presented, are all the source data underlying the results available to ensure full reproducibility?

No

Reviewer Expertise:

Social Science, International Relations, International Security, Border Security

I confirm that I have read this submission and believe that I have an appropriate level of expertise to confirm that it is of an acceptable scientific standard, however I have significant reservations, as outlined above.

# Reviewer report: Reviewer response for version 1

**DOI:** 10.21956/openreseurope.16307.r32648

**Published:** 2023-07-06

**Authors:** Andrew W Neal

**Affiliations:** 1 The University of Edinburgh, Edinburgh, Scotland, UK

This article provides a comprehensive scientific review of various terms related to participatory models, demonstrating a sophisticated and up-to-date understanding of the terminology. However, it is not entirely evident that this terminology posed a problem requiring immediate attention and rectification. While the review is valuable in expanding our knowledge of participatory models, a more explicit examination of the political applications and implications of these terms in the context of migration, security and human rights would enhance the article's overall contribution.

 The discussion on different perspectives concerning borders was noteworthy for its depth and nuance, particularly around borders as a process and performance. It shed light on the complex dynamics that shape the notion of borders in contemporary society; the authors provide valuable insights into the multifaceted nature of these boundaries. However, the arguments presented regarding surveillance and security as concepts are less convincing. While the authors contend that these concepts lack absolute values, I would argue that few if any concepts in political contexts possess absolute values. A more robust analysis is needed to elucidate how these essentially contested concepts intersect with the proposed participatory model and shape the dynamics of border control and surveillance.

 To advance the discussion, the focus should shift from a narrow understanding of actors, stakeholders, and end-users to an examination of how these groups compete in shaping the legitimacy and delegitimization of ‘Surveillance-Oriented Security Technologies’. By exploring the power struggles and competing interests within these dynamics, the authors could provide a more comprehensive understanding of the complexities surrounding participatory approaches in the context of security and surveillance.

 The authors rightly highlight the antagonistic relationship between ‘Surveillance-Oriented Security Technology’ actors and human rights organizations and civil society. This dynamic reflects the contentious politics surrounding migration, security, human rights, and humanitarian concerns. Incorporating an understanding of this antagonism into the proposed participatory model would offer a more realistic representation of the challenges and tensions inherent in these systems. Furthermore, the Frontex Consultative Forum on Fundamental Rights stands as a prominent example of this issue, and the omission of analysis represents a missed opportunity. Exploring the challenges, tensions, and power dynamics within this forum would enrich the article's analysis and contribute to a more comprehensive understanding of participatory models in the context of migration and security. See for example Perkowski, 2018 ^1^.

 The article tends to employ euphemistic language when discussing the ‘shortcomings’ in the management of EU migration and borders that came to light in the mid-2010s. A more explicit breakdown of these ‘shortcomings’, their causes, and particularly their consequences would provide a clearer understanding of the problems at hand. By delving into the intricacies of these shortcomings, such as political pressures, insufficient resources, and systemic failures, the authors could more effectively highlight the significance of addressing these issues. The authors do not acknowledge that lives of migrants, the international reputation of EU member states, and the authority and credibility of the international refugee and human rights regime are all significantly impacted by these matters. Such an expanded analysis would deepen our understanding of the stakes involved and the urgent need for comprehensive solutions.

 In conclusion, while this article offers a thorough exploration of certain terms related to participatory models, there is room for improvement in the overall argument and conception. A more explicit and comprehensive consideration of the implications of surveillance and security in migration control, along with an examination of relevant examples like the Frontex consultative forum, would enhance the article's impact. Additionally, a clearer and more direct engagement with the significant stakes involved in these issues would contribute to a deeper understanding of their importance in the context of migration, security, human rights, and humanitarian concerns. By incorporating these enhancements, the article could make a more substantial and compelling contribution to the field.

Are sufficient details provided to allow replication of the method development and its use by others?

Partly

Is the description of the method technically sound?

Partly

Are the conclusions about the method and its performance adequately supported by the findings presented in the article?

Partly

Is the rationale for developing the new method (or application) clearly explained

Partly

If any results are presented, are all the source data underlying the results available to ensure full reproducibility?

No source data required

Reviewer Expertise:

Security politics and critical security studies, including migration, democratic politics, securitisation and theory.

I confirm that I have read this submission and believe that I have an appropriate level of expertise to confirm that it is of an acceptable scientific standard, however I have significant reservations, as outlined above.

## References

[1] : Frontex and the convergence of humanitarianism, human rights and security. *Security Dialogue* .2018;49(6) : 10.1177/0967010618796670 457-475 10.1177/0967010618796670

# Reviewer report: Reviewer response for version 1

**DOI:** 10.21956/openreseurope.16307.r30714

**Published:** 2023-02-28

**Authors:** Bartosz Czepil, Wojciech Opioła

**Affiliations:** 1 Institute of Political Science and Public Administration, University of Opole, Opole, Poland; 2 Department of Regional Studies, University of Opole, Opole, Poland

This article refers to a very important problem, which will be faced by governments and international organizations with growing intensity, because of unprecedented international migratory flows. This is the problem of securing borders without breaching of human rights (or with protection of human rights) when security policies are implemented and a new surveillance technologies are being used. So, studying this problem and providing solutions for approaching migration/security/human rights nexus is valuable undertaking which deserves scholarly attention. However, being convinced that the research work of the Authors is important one, I have (together with co-reviewer) several remarks towards different aspects of the article.

 I think that justification for building the APM could be stronger. The Authors refer rightly to the international organizations’ (IOM, UNHCR, EU) postulate to integrate borders management with human rights protection, articulating ‘interlaced’ nature of these two dimensions. These organizations point to the necessity of inclusion HRGs and CSOs into process of border management. But I do not see any reference to problems faced by civil society sector (including CSO and HRG) when trying to influence and participate in approaching/shaping border security policies by governmental agencies. In my opinion these two sectors (CSO/HRGs and governments/security agencies together with political parties having control over these institutions) have very often contradictory priorities, values and interests, sometimes clashing over an issue of border control. This was clearly visible during the migration crisis on the Polish-Belarusian border in 2021-2022, when humanitarian organizations and media had limited assess to the border area because the state of emergency was imposed along the border with Belarus. Generally speaking, HRGs and CSOs very often expose wrongdoings (for example push-backs) of governmental agencies implementing security policies, they present critical stance towards governments, while governments criticize them depicting their actions as irresponsible security threat. I think that this conflicting interests and perceptions have been neglected throughout the whole article.

 Secondly, I think that the steps towards building the ARESIBO Participatory Model are not fully relevant and transparent. The Authors conducted very sophisticated desk research based on two stages, each guided by three research questions. Moreover, the data gathered were analysed on three levels. This was aimed at finding out how the concepts like stake-holders, actors and end-users are understood in general. I am still not sure why is this knowledge relevant and why does it demand such sophisticated and, I believe, time consuming data analysis? These are commonly used concepts in social science and in the sector of project implementation. Additionally, they strongly overlap (this is one of the obvious findings of the desk research), so they might be confusing when selecting and naming targets of the involvement. Thus, I would not put them as such important part of the model building. Additionally, the 3 levels of analysis distinguished by the authors are confusing for me. The 3 ^rd^ level includes, according to my understanding, academic research which is a very essence of the 1 ^st^ level – why is it so? Additionally the ‘EU funded research’ have been included into 3 ^rd^ level as if these were not academic research (studied on the 1 ^st^ level). I think that the Authors do not follow this three-level distinction consequently, because in the Results part (for example p. 7 and p. 9) they study the ‘EU funded research’ named as the 1 ^st^ level, while studying academic research sources is named as the 2 ^nd^ level. Thus, with due respect to the Authors, I think that the trivial conclusions have been achieved through sophisticated, however not fully transparent desk research. Moreover, the desk research conducted was aimed at analysis of commonly used concepts without any attempt to connect them with the essential problems articulated at the very beginning of the article – the involvement of HRGs and CSOs into process of using SOST when managing borders. I would rather see thorough review of problems, good practices and bad experiences of CSOs and HRGs dealing with human rights issues, both when it comes to the border management and using of surveillance technologies – this would constitute sound base for building the proposed model.

 Last but not least, the issue of proposed model (APM). I think that it is a very general, abstract and not sufficiently embedded in peculiarity of the problems declared at the very beginning of the article. I am aware that a model is an abstract, theoretical framework from its very nature, but I would rather expect some theoretical proposal which takes into account a specific context of border management, surveillance technologies and the position of HRG and CSO within this context. It would be valuable to write more about experience from testing the model as the Authors inform that the model was tested (p. 12). For this moment we have rather a general proposal which might be applied to every process/organization demanding involvement of some third parties (target groups). I do not see how it addresses the main challenges/barriers/opportunities with involvement of civil society into process of using surveillance technologies in the border control. For example it does not take into account the different interests of civil society sector and governmental actors mentioned already. In my opinion, before we start to think about an impact, co-design, co-production we need, first of all, think of modelling mechanism supporting/motivating to cooperation between different actors/stakeholders. What is interesting for me is that after such sophisticated work on defining actors, stakeholders and end-users, we still do not know which of these categories refers to HRG and CSO. Actually, the taxonomy of targets is still not clear because citizens, might be treated as actors, end-users and stake-holders. Are HRGs and CSOs actors, stakeholders or citizens? There is no any clear description/enumeration of specific surveillance technologies used on the border (the Authors mention about drones and biometrics) and how exactly we can involve HRGs and CSOs into its usage on the ground. The Authors claim (in the APS part) that involvement into SOST development process will reflect the main good governance principles (participation, responsiveness, efficiency etc.), but this seems to be rather a moral postulate now, because I do not see what kind of solutions are designed to ensure this principles. Summarizing, in my humble opinion, the APM is too detached from the problems and goals declared at the beginning of the paper and I would not know how to apply it to a specific border area when trying to improve participation of HRGs and CSOs in border security management. However, I believe that building such model and studying the nexus between border security, migration and human rights is important research and policy problem, which makes the proposed article valuable undertaking.

Are sufficient details provided to allow replication of the method development and its use by others?

Partly

Is the description of the method technically sound?

Partly

Are the conclusions about the method and its performance adequately supported by the findings presented in the article?

No

Is the rationale for developing the new method (or application) clearly explained

Partly

If any results are presented, are all the source data underlying the results available to ensure full reproducibility?

No source data required

Reviewer Expertise:

Quality of governance, corruption and anti-corruption, border and regional studies

We confirm that we have read this submission and believe that we have an appropriate level of expertise to confirm that it is of an acceptable scientific standard, however we have significant reservations, as outlined above.
